# Financial Incentives to Promote Active Travel: An Evidence Review and Economic Framework

**DOI:** 10.1016/j.amepre.2012.09.001

**Published:** 2012-12

**Authors:** Adam Martin, Marc Suhrcke, David Ogilvie

**Affiliations:** a Health Economics Group, Norwich Medical School, University of East Anglia, Norwich, United Kingdom; b MRC Epidemiology Unit, Cambridge, United Kingdom; c UKCRC Centre for Diet and Activity Research, Institute of Public Health, Cambridge, United Kingdom

## Abstract

**Context:**

Financial incentives, including taxes and subsidies, can be used to encourage behavior change. They are common in transport policy for tackling externalities associated with use of motor vehicles, and in public health for influencing alcohol consumption and smoking behaviors. Financial incentives also offer policymakers a compromise between “nudging,” which may be insufficient for changing habitual behavior, and regulations that restrict individual choice.

**Evidence acquisition:**

The literature review identified studies published between January 1997 and January 2012 of financial incentives relating to any mode of travel in which the impact on active travel, physical activity, or obesity levels was reported. It encompassed macroenvironmental schemes, such as gasoline taxes, and microenvironmental schemes, such as employer-subsidized bicycles. Five relevant reviews and 20 primary studies (of which nine were not included in the reviews) were identified.

**Evidence synthesis:**

The results show that more-robust evidence is required if policymakers are to maximize the health impact of fiscal policy relating to transport schemes of this kind.

**Conclusions:**

Drawing on a literature review and insights from the SLOTH (sleep, leisure, occupation, transportation, and home-based activities) time-budget model, this paper argues that financial incentives may have a larger role in promoting walking and cycling than is acknowledged generally.

## Context

During the past century, most developed countries have witnessed a considerable rise in the prevalence of obesity.^1^ A dominant view among economists is that this trend is attributable largely to a utility-maximizing response of individuals to technologic progress that has decreased the price of energy intake (via reduced food prices) and increased the price of energy expenditure (via growing opportunity costs of physical activity).^2^ Table 1 shows the impact of these changes on the costs people face when making decisions about physical activity and food consumption during their daily leisure, work, travel, and home-based activities. For example, technologic innovation in agriculture, food production, and retail has contributed to reduced costs (including time costs) of energy-dense meals, and working environments typically have become more office-based and sedentary.

The present paper is concerned primarily with the impact on decision making of changes in the cost of travel. Travel is a hitherto relatively under-exploited area for promoting health behavior change, but is potentially important in the “small changes approach” to tackling obesity, which focuses on small but achievable improvements in physical activity rather than more-substantial lifestyle changes that have sometimes proven unrealistic.^3^ Because cycling and walking can be integrated more readily into people's busy schedules than, for example, leisure-time exercise,^4,5^ these could represent low-cost, acceptable, and accessible ways to achieve 30 minutes of daily, moderate-intensity physical activity as recommended in international guidelines to help prevent obesity and more than 20 other chronic conditions.^6–10^

More specifically, the current paper explores the potential for financial incentives to encourage physical activity through active travel and influence related health outcomes. Financial incentives are policies involving a targeted payment to, or withdrawal of monetary resources from, an individual's budget. They encompass interventions at the macroenvironmental (e.g., government) and microenvironmental (e.g., workplace) levels,^11^ including positive financial incentives^12^ rewarding active travel and negative financial incentives penalizing sedentary travel.

## Evidence Acquisition

### Identification of Relevant Studies

The review identified studies of financial incentives relating to any mode of travel in which the impact on active travel, physical activity, or obesity levels was reported. The ECONLIT, Google Scholar, National Bureau of Economic Research (NBER) and PubMed electronic databases were searched between May 2011 and January 2012 with terms relating to “physical activity,” “transport,” “built environment,” and “prices.” Non-English-language papers, and studies published before 1997, were excluded. Five relevant reviews and 20 primary studies (of which nine were not included in the reviews) were identified (Table 2).

### Data Extraction and Quality Assessment

Information was extracted on study place and year; study design; intervention and population characteristics; and results. Quality assessment focused on the likelihood that causal inferences may be drawn,^13^ based on a method originally devised for use in criminology reviews.^14^

## Evidence Synthesis

### Description of Studies

The majority of studies (70%) presented evidence for a particular microenvironmental scheme. Together, only a small range of schemes were represented, predominantly involving free bicycles or local road pricing at specific locations and generally within particular population subgroups. The majority (67%) of intervention studies used uncontrolled cross-sectional analysis of population-level data, which cannot support robust causal inference. Further, most considered only changes in travel behavior or physical activity (87%), so improvements in health or reductions in obesity only can be estimated. Higher-quality study designs used included RCTs (20%), although, as with other the intervention studies, these often had short follow-up periods (average 7 months).

### Positive Financial Incentives

Five recent reviews^15–19^ that included microenvironmental interventions to promote active travel identified just three examples of positive financial incentives, all involving free bicycles. One RCT^20^ involving Swedish women with abdominal obesity reported a significant increase in the proportion of women cycling more than 2 km per day after 18 months. Two uncontrolled studies^21,22^ found that the Danish “Bikebusters” and the Australian “Cycle100” schemes led to significant increases in the proportion of trips made by bicycle (from 9% to 28% in “Bikebusters”), although both involved selected participants.

Additional evidence, not captured in the five reviews, included an RCT^23^ involving 51 older Americans in which significant differences in average daily “aerobic minutes” were identified between a group receiving fixed weekly payments of $75 and a comparison group receiving $50 plus $10 (or $25) contingent on averaging at least 15 (or 40) aerobic minutes per day each week. “Aerobic minutes” were measured using pedometers and defined as continuous walking (not necessarily for transport), jogging, or running at a rate above 60 steps per minute for at least 10 minutes. Two further studies^24,25^ reported stated preference data. One^25^ of these showed that a £2 daily payment to cyclists could increase cycling by 88%, although these studies relied on individuals choosing between hypothetic alternatives.

Many studies in transport economics have shown a negative price elasticity of demand for public transport,^26^ indicating that price reductions would lead to increased demand. If, as three studies^27–29^ show, this displaces car journeys (rather than active travel), then increased physical activity would be expected because public transport use typically is accompanied by some walking.^30–33^ At the microenvironmental level, in the first study,^27^ an RCT reported significant increases in the proportion of people using public transport (from 18% to 47%) and reductions in car use (from 50% to 33%) in an intervention group that received free public transport passes in Stuttgart, Germany. Respective changes in the control group were not significant and there were no changes in cycling or walking trips. In the second study,^28^ higher employee physical activity levels were shown in U.S. workplaces that provided subsidized public transport passes compared to those that did not. However, the effect may have been over-estimated because workplaces were more likely to provide a subsidy if public transport facilities were within walking distance.

At the macroenvironmental level, the impact of free bus passes, available to older people in England since 2006, was examined using a logistic regression analysis of the English Longitudinal Study of Ageing (ELSA).^29^ Eligibility for the free pass was associated with a 51% increase in the odds of using public transport, whereas public transport use in old age was associated with 21% lower odds of being obese, even after adjustment for previous weight status. A fourth study,^34^ of free bus passes available to young people in London, England, since 2008, showed that although increased public transport demand displaced some active travel journeys, physical activity increased because the pass generated more journeys overall.

### Negative Financial Incentives

At the microenvironmental level, one review^35^ identified limited evidence from two intervention studies about the impact of road-user charging on physical activity. In Durham, England,^36^ a 10% increase in pedestrian activity was reported 1 year after the scheme started, and in London,^37^ distances cycled increased by 30% over a 3-year period.

In Zoetermeer, The Netherlands, a study showed that 14% of car drivers switched to alternative travel modes after daily financial incentives of €3 to €7 were given to regular commuters in return for avoiding specific road sections.^38,39^ In Stockholm, Sweden, another study^40^ found a 25% reduction in the number of car journeys in response to a temporary $2 congestion charge. Small increases in public transport use and self-reported physical activity levels also were identified. In Trondheim, Norway, one study^41^ attributed an increase in car journeys and decreases in public transport use, cycling, walking, and car occupancy to the withdrawal of road pricing.

Other microenvironmental evidence includes a study^42^ reporting a threefold increase in cycling among employees at Manchester Airport, England, attributed to a Workplace Travel Plan that included increased car parking charges, and other reports^43^ that those Workplace Travel Plans which included car-sharing financial incentives had the greatest chance of reducing car use. A further study^44^ of eight California workplaces reported a 39% increase in active commuting attributable to “cashing out,” in which individuals receive payment for not using their free workplace car parking space. However, these three studies were poorly controlled and the changes were small in absolute terms.

At the macroenvironmental level, two studies^45,46^ identified a significant inverse relationship between gasoline prices and obesity prevalence (defined as the proportion of individuals with a BMI ≥30). The first^45^ drew cross-national comparisons of 24 European countries. Using U.S. data, the second^46^ suggested that 8% of the rise in obesity prevalence between 1979 and 2004 was attributable to declining gasoline prices (via reduced walking and increased restaurant visits). It implied that a $1/gallon gasoline tax would reduce obesity prevalence by 10%, with some evidence that women, ethnic minorities, and lower-income groups were most responsive to price changes (although this may have been due to their living in urban areas with public transport facilities).

One study^47^ involving 20 years' worth of cohort data from 5115 U.S. individuals demonstrated a positive association between gasoline prices and physical activity. Roughly, there were 17 minutes of additional walking each week after a $0.25 per gallon increase. The study also suggested that the price change might encourage individuals to replace physical activity away from home (e.g., bowling) with activities in the immediate area (e.g., jogging).

Econometric analysis also has been used to show an inverse relationship between gasoline taxation and gasoline consumption.^48^ One review^49^ estimated that a 10% rise in gasoline prices was associated with reductions of 3% in road traffic and 2.5% in car ownership. Although more active travel cannot be inferred, because car trips are less responsive to gasoline prices than fuel consumption and distance traveled,^50^ some studies did report a positive relationship between gasoline prices and demand for other travel modes.^49^ For example, one U.S. study^51^ used self-reported data from a national survey to claim that cycling increased by 4.7% for men and 3.5% for women after a $1 per gallon gas price increase.

### Summary

This review identified only a limited amount of evidence on financial incentives for active travel. Although the identified studies provide useful insights into specific interventions for particular populations, a more general understanding about how people might be expected to respond has yet to emerge.

## Discussion

One partial explanation for the shortage of empirical evidence, particularly at the macroenvironmental level, may be the potential political risks generally associated with financial incentives.^15,52,53^ Negative financial incentives typically require strong justification because they penalize individuals who happen to have made particular choices, whereas positive financial incentives require substantial financial investment.^54,55^

**Figure 1:**
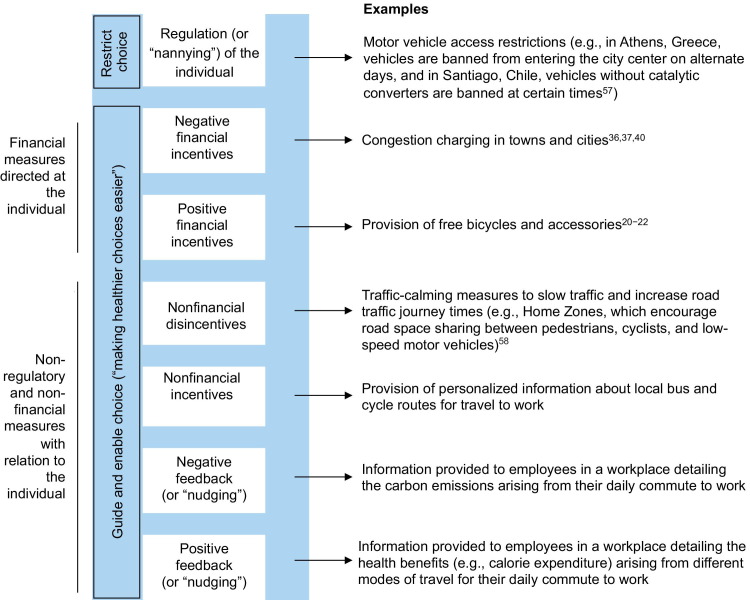
A hierarchy of policy interventions to support active travel *Note:* Higher rungs on the ladder represent decreasing acceptability and increasing intrusiveness (as suggested in the Nuffield Intervention Ladder^55^). Decision makers should only consider policies on higher rungs of the ladder if policies on lower rungs are deemed to be ineffective.

However, financial incentives for active travel could be viewed somewhat more favorably as they fall neatly between regulating (or “nannying”), which is sometimes regarded as overly restricting choice, and interventions that provide feedback (or “nudging”), which might not be highly effective when used in isolation^56^ (Figure 1). They also could reinforce existing government priorities such as environmental sustainability, tackling health inequalities, and economic growth (via reduced congestion and absenteeism). Further, implementation may prove relatively straightforward if integrated somehow with existing transport schemes designed to internalize externalities including congestion, injuries, pollution,^59^ and even risky driving.^60^ Relevant lessons also might be drawn from financial incentives used in health care to reduce smoking, alcohol, and obesity^61^; improve patient compliance^62^; and encourage Chlamydia screening.^63^

To gain a more comprehensive understanding of the complex individual-level impact of financial incentives on travel behavior and health, higher-quality studies that support more-robust causal inference are required. Reliance on uncontrolled cross-sectional studies with short follow-up periods particularly limits the potential for understanding downstream changes, such as body size, or how to prevent people from returning to old habits after financial incentives are withdrawn.^12,15,64^ Such studies also may have limited external validity if they include only small population subsets, such as ethnic minority, low-income groups in high-density urban areas (one study shows that walking to public transport is especially common in these groups),^30^ or people who have recently moved.^27,65^ Further, biased effect estimates can occur if the quality of the built environment, which may support or hinder active travel,^66,67^ or other factors, such as climate or the supportiveness of employers, are not controlled for.

Although RCTs may sometimes be unrealistic or politically untenable,^68^ “natural experiment” designs, in which a “natural or predetermined variation of allocation occurs,”^69,70^ provide a promising alternative. These include intervention studies with large individual-level data sets, such as those proposed for the evaluation of various policy and infrastructure projects in the United Kingdom,^34,71,72^ and non-intervention studies relating particularly to negative financial incentives, which rely mainly on observed relationships between population-level behavior and price changes over time. Although the latter provide a weaker basis for causal inference, similar econometric evidence supported the initial case for tobacco taxation.^73^ With appropriate data, these methods also can contribute to a deeper understanding of the distribution of health benefits across various population groups and provide important insights into the types of financial incentives most likely to deliver long-term behavior change.

### Other Insights from Economic Rational-Choice Frameworks

Appendix A describes how an economic rational-choice framework might be developed to draw some broader insights into people's likely responses to financial incentives for active travel. It incorporates elements of the SLOTH time-budget model,^74–76^ and Lakdawalla-Philipson's utility maximization model,^77^ developed elsewhere for analyzing the multitude of decisions people make when allocating scarce resources of time and money to competing demands. This analysis provides a useful illustration of two broad points that were not established in the literature review and are in some contrast to existing SLOTH-based analyses which suggest that “leisure becomes the most likely area for increasing physical activity”^76^ because (for simplicity) the trade-offs associated with leisure and travel decisions have been treated as though identical.

First, the framework suggests that individuals are likely to be at least as (if not more) responsive to financial incentives for active travel as those for active leisure, a view reflected in recent panel data analysis that shows active leisure “comes and goes” and “exercise as part of travel and work must be emphasized.”^78^ Second, active travel allows people to access work and leisure activities but, unlike sedentary travel, is also “productive” in the sense of enabling energy expenditure. Yet established methods for transport appraisal place large monetary values on travel-time savings to justify investment in transport infrastructure on the basis that (for travel in work hours) savings in travel time convert nonproductive time to productive use.^79–81^ In contrast to car travel, others have argued that this overlooks the potential to use rail travel productively for work activities.^82,83^ Similarly, these methods probably favor faster sedentary travel (cars and trains) over active travel, despite active travel being suitable for most journeys.^84^

These methods also may have encouraged decline in the availability of local services that are particularly accessible by active travel. In the United Kingdom, where travel-time savings have accounted for around 80% of the claimed monetary benefits of major road schemes, the average time that people spend traveling has remained constant since the 1960s.^85^ This suggests that motorway (freeway) expansion has encouraged long-distance travel for access to work and leisure opportunities much farther from home. People who choose active travel may then experience mobility-related social exclusion,^82^ where they are disadvantaged in terms of access to services.

In the absence of more empirical evidence, further development of a modeling approach to active-travel decisions may prove advantageous; however psychological theories of behavior and recent empirical work in behavioral economics should be incorporated alongside standard rational behavior assumptions.^86–88^ For example, overly self-focused behavior,^89^ strong habitual behavior, optimism bias, and ingrained social norms may all favor motorized transport and discourage individuals from giving rational consideration to active travel modes.^90^ The resulting “car dependency” may be reinforced by car manufacturers through marketing and political lobbying.^91^

**Figure 2:**
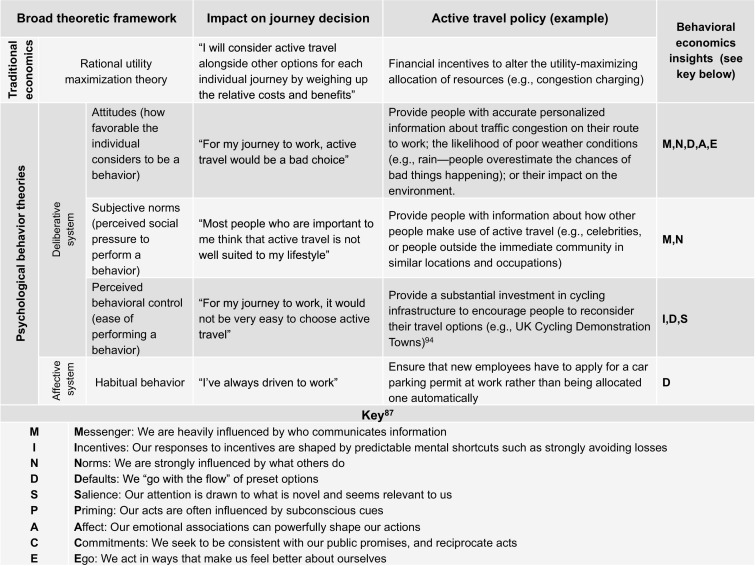
Alternative theoretic perspectives on travel mode choices and active travel policies

These factors, and policies for moderating them, are explored in Figure 2 in the context of the theory that individual behavior is determined by a deliberative system, which assesses options with a broad, goal-based perspective, and an affective system that encompasses emotions and motivational drives.^92^ The deliberative system is described in Ajzen's theory of planned behavior as comprising attitudes, subjective norms, and perceived behavioral control.^93^ For example, the Cycling Demonstration Towns program in England, in which per capita investment in schemes to promote cycling was increased in six urban areas to ten times the national average,^94^ might be viewed as a method of influencing habitual behavior (“changing the default”) and “status quo bias,” where people tend to maintain established behaviors unless incentives to change are substantial. However, studies specifically examining the impact of financial incentives on habitual travel behavior have produced inconclusive results.^27,95^

In addition to habitual behavior, excessive driving also might occur because people feel they ought to drive more often in order to justify the high sunk (i.e., retrospective and nonrecoverable) costs they incurred when buying a car. Like rail commuters with annual season tickets,^96^ they find that additional journeys incur low marginal costs. Yet, when encouraged to consider only the (smaller) average cost of each journey, the utility-maximizing allocation of resources would involve more active travel.

Although the evidence is limited, “car clubs,” in which car drivers hire cars for short periods rather than owning them outright, are reported to have reduced car mileage (by 33% in The Netherlands),^97^ increased cycling,^98^ and reduced motor vehicle ownership.^99^ Bicycle hire schemes might have a similar impact in the sense that car drivers are not deterred by the monetary and other costs (e.g., those arising from unfamiliarity) of a bike purchase. In the Netherlands, a before-and-after study has attributed reductions in car use and increases in cycling to such schemes.^100^ Public transport “clubs,” which encourage passengers to consider marginal (rather than average) costs by making a large upfront payment for future discounted public transport tickets, also have encouraged higher tram and bus use in some Swiss cities,^101^ although any association with fewer car journeys is unknown.

### Conclusion

Recent empirical evidence, complemented by a simple economic rational-choice framework, suggests that financial incentives for active travel may represent an underused but potentially promising method for encouraging healthier behaviors. However, higher-quality studies, particularly at the macroenvironmental level, are required if policymakers are to use evidence of effectiveness to make confident decisions about allocating scarce resources to such schemes.

## Figures and Tables

**Table 1:** Examples of the impact of technologic progress on the costs of energy intake and energy expenditure

Activity domain	Costs of energy expenditure		Costs of energy intake
	Increasing opportunity costs of energy expenditure	Increasing monetary costs of energy expenditure	Decreasing costs of food consumption
Sleep	N/A (The time spent sleeping has remained broadly constant)		
Leisure	Greater opportunity for sedentary leisure activities (e.g., TV, computers, and the Internet)	Greater availability of active leisure facilities away from home that incur a financial cost (e.g., leisure centres, swimming pools, and gyms)	Increased availability of restaurants (including fast-food)
Occupation	Greater availability of, and higher wages associated with, sedentary work	The change from an agricultural or industrial society means that, in a sense, people are no longer paid to exercise at work.	Greater availability of mass-produced, energy-dense, packaged, snack foods which can be consumed “on the go” (and are often heavily marketed, perhaps appealing to a lack of self-control and hyperbolic discounting which apparently characterizes food consumption)
Transportation	Availability of motorized transport and investment in road networks has provided greater opportunities for faster and longer-distance journeys which are not well suited to active travel modes	N/A	Expansion of “Drive-Thru” takeaway services which allow consumption of fast-food while traveling
Home	Modern technology (e.g., gardening tools and kitchen appliances) allows household chores to be done more quickly with less physical effort	N/A	Transfer of labor-intensive food preparation to intensive farming, supermarkets, and factories, has dramatically reduced the costs (including time costs) associated with food preparation at home. The availability and quality of kitchen appliances such as microwaves, refrigerators, and freezers also have improved.

N/A, not applicable

**Table 2:** Summary of evidence relating to financial incentives identified in the review

REVIEWS		
Review reference	Review	Title
A	Mackett (2011)^15^	Transport, physical activity, and health: present knowledge and the way ahead
B	Ogilvie (2004)^16^	Promoting walking and cycling as an alternative to using cars: systematic review
C	Ogilvie (2007)^17^	Interventions to promote walking: systematic review
D	Pucher (2010)^18^	Infrastructure, programs, and policies to increase bicycling: An international review
E	Yang (2010)^19^	Interventions to promote cycling: systematic review

STUDIES												
Study [review reference]	Study design		Study description			Results						
	Study design description (checklist score^a^)	Intervention study	Country	Population	Description of intervention	Outcome	Comparator	Follow-up (months)	Reported outcomes			Individual-(I) or population-(P) level data
									Travel mode	Active travel or physical activity	Obesity, BMI or weight	
**POSITIVE FINANCIAL INCENTIVES**												
**Walking and cycling**												
Hemmingson (2009)^20^ [D,E]	RCT (7)	✓	Sweden	Middle-aged women with abdominal obesity	A moderate-intensity program including free bicycles	Significant increase in women cycling more than 2 km per day	Control group involving a low-intensity program (excluding free bicycles)	18	✓	✓	✓	I
Bunde (1997)^21^ [B,D]	Uncontrolled before–after study (0)	✓	Denmark	Adults	Free bicycles (“Bikebusters”)	Increase in proportion of trips made by bike (from 9% to 28%)	Proportion of trips made by bike before the intervention	11	✓	✓		P
Bauman (2008)^22^ [A]	Uncontrolled before–after study (0)	✓	Australia	Adults	Free bicycles (“Cycle 100”)	Increase in proportion of trips made by bike	Proportion of trips made by bike before the intervention	Not reported	✓	✓		P
Finkelstein (2008)^23^	RCT (7)	✓	U.S.	Older adults	Payments contingent on exercise levels (number of “aerobic minutes”)	Significant differences in exercise levels	Individuals who receive a fixed payment irrespective of exercise levels	1	✓	✓		I
Ryley (2006)^24^; Wardman (2007)^25^	Stated Preference Data (N/A)		United Kingdom	Adults	Hypothetic payment to individuals in return for cycling more often	In one case, an increase in proportion of trips made by bike of 88%	Hypothetic case where payments are not made to individuals	N/A	✓	✓		I
**Public transportation**												
Bamberg (2006)^27^ [A]	RCT (7)	✓	Germany, Stuttgart	People who have recently (within 6 months) moved to the city	Subsidized public transport passes	Significant increases in the proportion of people using public transport and reductions in car use	Before and after the intervention (in the intervention group) and compared to respective analysis in the control group	1.5	✓	✓		I
Lachapelle (2009)^28^ [A]	Observational study (0)	✓	U.S.	Workplace employees	Subsidized public transport passes	Significant increases in physical activity levels	Workplaces that do not offer subsidized public transport passes	N/A (cross-sectional study)	✓	✓		P
Webb (2011)^29^	Controlled study with analysis of change at individual level (4)	✓	England	Older people	Subsidized public transport passes	Free pass was associated with increased public transport use. Public transport use was associated with lower obesity	Logistic regression analysis using panel data	24	✓	✓	✓	I
Jones (2012)^34^	Qualitative observational study (0)	✓	England, London	Young people	Subsidized public transport passes	Physical activity increased since young people reported an increase in journeys made	Young people's own accounts of bus travel arising from interviews and focus groups	N/A	✓	✓		I
**NEGATIVE FINANCIAL INCENTIVES**												
**Walking and cycling**												
Durham Council (2006)^36^ [A]	Uncontrolled before–after study (0)	✓	England, Durham	Drivers	Road pricing	A 10% increase in pedestrian activity	Before the road pricing was introduced	9	✓	✓		P
Transport for London (2006)^37^ [A]	Uncontrolled before–after study (0)	✓	England, London	Drivers	Road pricing	Distances cycled increased by 30%	Before the road pricing was introduced	36	✓	✓		P
Ben-Elia (2011)^38^; Bliemer (2010)^39^	Uncontrolled before–after study (0)	✓	The Netherlands, Zoetermeer	Car drivers	Financial incentives of $3 to $7	14% of drivers switched to alternative travel modes	Individual behavior before the financial incentive was introduced	3	✓			I
Bergman (2010)^40^ [A]	Uncontrolled before–after study (0)	✓	Sweden, Stockholm	Car drivers	$2 congestion charge	25% reduction in number of car journeys	Before the road pricing was introduced (and comparisons with similar cities to suggest a real effect attributable to the policy)	30	✓	✓		P
Meland (2010)^41^ [A,B]	Uncontrolled before–after study (0)	✓	Norway, Trondheim	Car drivers	Removal of a road pricing system	Increased car journeys and decreases in public transport and active travel	Before the withdrawal of road pricing	Up to 12	✓	✓		P
Shoup (1997)^44^ [B,D,E]	Uncontrolled before–after study (0)	✓	U.S., California	Car drivers (commuters)	Payment for not using a car park	39% increase in active commuting	Before the scheme	Up to 36	✓	✓		P
Rye (2002)^42^ [D]	Uncontrolled before–after study (0)	✓	England, Manchester Airport	Car drivers (commuters)	Car park charging (as part of a Work Place Travel Plan)	A threefold increase in cycling	Before the scheme	Not reported	✓	✓		P
**Gasoline prices**												
Rabin (2007)^45^	Cross- sectional, observational study using linear regression (0)		24 European countries	Country-level data	None	Significant inverse relationship between obesity levels and obesity prevalence	Cross-national comparisons are made	N/A (Cross-sectional study)	✓		✓	P
Courtemarche (2011)^46^	Individual- level repeated cross-sectional study (0)		U.S.	Adults	None	Significant inverse relationship between obesity levels and obesity prevalence	Changes in gas prices over time	20 years	✓	✓	✓	I
Hou (2011)^47^	Random-effect longitudinal regression using individual-level data (3)		U.S., four cities	Young adults (aged 18–30 years at baseline)	None	Significant relationship between gas prices and physical activity	Changes in gas prices over time (the individuals act as their own controls)	15 years	✓	✓	✓	I
Rashad (2009)^51^	Cross- sectional multivariate regression analysis (0)		U.S.	Adults	None	Significant relationship between gas prices and self-reported cycling	Comparison of individuals in different areas with different gas prices	N/A (Cross-sectional study)	✓	✓		I

N/A, not applicable

a A higher score on the checklist represents increasing likelihood that causal inferences may be drawn. 0 = study designs from which causal inferences cannot be drawn; 1–4 = study designs from which some causal inferences may be drawn depending on the extent to which there is analysis of change over time and whether (observable and unobservable) characteristics are controlled for; 5–7 = study designs most likely to support robust causal inferences (5–6 = randomization in a natural-experiment setting; 7 = randomization in an controlled-experiment setting).
