# Supplementary material for: Financial Incentives to Promote Active Travel: An Evidence Review and Economic Framework
Source: Am J Prev Med. 2012 Dec;43(6):e45–57. doi: 10.1016/j.amepre.2012.09.001 (PMC3834139; doi:10.1016/j.amepre.2012.09.001)
Supplement: Appendix A [file mmc1.pdf]

Financial Incentives to Promote Active Travel

An Evidence Review and Economic Framework

Adam Martin, MSc, Marc Suhrcke, PhD, David Ogilvie, PhD

Appendix A

This appendix describes an economic rational-choice framework that incorporates elements of the SLOTH (sleep, leisure, occupation, transportation, and home-based activities) time-budget model,[1–3] and Lakdawalla-Philipson's[4] utility-maximization model, developed elsewhere for analyzing decisions people make when allocating scarce resources of time and money to competing demands. A number of simplifying assumptions have been made (summarized in Table A1), and more-detailed analysis might be possible using a full economic model similar to Yaniv's work[5] on healthy-eating financial incentives. However, to complement the specific examples identified in the evidence review, the framework is designed to provide broader insights into people's likely responses to financial incentives for active travel in a way that contrasts with existing SLOTH-based analyses, which suggest that "leisure becomes the most likely area for increasing physical activity"[3] because the trade-offs associated with leisure and travel decisions are treated as though identical.

Resource Constraints (Y and Z)

Individuals are subject to:

a time constraint (Z hours in the current period) such that time is allocated to SLOTH activities and, within each domain, to sedentary or physically active behaviors:

$$\mathbf{S} + \mathbf{L} + \mathbf{O} + \mathbf{T} + \mathbf{H} = \mathbf{S}\mathrm{s} + \mathbf{L}\mathrm{s} + \mathbf{L}\mathrm{p} + \mathbf{O}\mathrm{s} + \mathbf{O}\mathrm{p} + \mathbf{T}\mathrm{s} + \mathbf{T}\mathrm{p} + \mathbf{H}\mathrm{s} + \mathbf{H}\mathrm{p} = \mathrm{Z} \qquad \text{(I)}$$

(where bold letters indicate time allocated to domain-based activities; lower-case s stands for sedentary activity; p stands for physically active behaviors);

an income constraint such that expenditure ($ per unit of time) on leisure ($L [e.g., cost of pool entrance for swimming]); transport ($T [e.g., cost of a train ticket]); and home ($H [e.g., cost of cooking ingredients]) cannot exceed income (Y):

$$\$\mathrm{L} + \$\mathrm{T} + \$\mathrm{H} = \mathrm{Y} \qquad \text{(II)}$$

Income (Y) is determined by time allocated to work and the wage rate (w, $ per unit of time):

$$\mathrm{Y} = \mathbf{O} \times \mathrm{w} \qquad \text{(III)}$$

Utility Maximization

An individual's current-period utility depends on consumption of sleep, leisure, home and transport activities (S, L, T, and H); weight in the current period (W); and their own valuation of their expected weight in the next period (βv [W']):

$$\mathrm{U(W)} = \mathrm{U}(\mathbf{S}, \mathbf{L}(\$\mathrm{L}, \mathrm{mL}), \mathbf{T}(\$\mathrm{T}), \mathbf{H}(\$\mathrm{H}), \mathrm{W}) + \beta\mathrm{v}\,(\mathrm{W'}) \qquad \text{(IV)}$$

[where U=utility; mL=distance (miles) travelled to leisure activity; $L/$H=leisure or home-based expenditure; W=current weight; W'=expected weight in next period; βv=discounted value of future weight]

$$W' = (1 - \delta)W + g(E, F) \quad \text{(V)}$$

[where δ <1; g is continuous, concave, increasing in food consumption (F) and decreasing in energy expenditure (E), as described in Lakdawalla-Philipson's utility-maximization model].[4]

Utility increases or decreases in weight, depending on whether the individual is above or below their (own notion of) "ideal weight" (W_0). They prefer weight gain when below W_0, and weight loss when above W_0. Future weight (W') is influenced by current-period choices about physical activity and food consumption (E and F). Energy expenditure (E) increases with domain-based physical activity (e.g., Lp) and is treated as a ratio of time allocated to physical relative to sedentary activities:

$$E = \frac{(Lp + Op + Tp + Hp)}{(Ss + Ls + Os + Ts + Hs)} \quad \text{(VI)}$$

Standard economic assumptions state that utility rises with consumption of S, L, and H at diminishing marginal rates and, for given L and H, increases with expenditure ($H and $L). Distance from home to any specific work (mO) or leisure (mL) activity (e.g., a person's own workplace, or their preferred gym) is fixed, because individuals cannot influence the locations of those destinations in the short-term. All else being equal, people seek to minimize travel distances, but will choose to travel further (higher mL) to access particular leisure activities that offer higher marginal utility than those available locally or at home (e.g., a park is chosen over gardening only if it provides higher utility). In the same way, individuals will choose to spend more money on an activity (e.g., swimming) only if it provides higher marginal utility than cheaper alternatives (e.g., gardening).

Choices about S, L, and H are determined by the "last hour" and "last dollar" rules which state that if the last hour or dollar invested in one activity (e.g., swimming) provides greater utility than the last unit invested in another (e.g., home cooking), then each day, individuals will reallocate resources in favor of activities that deliver higher returns (i.e., all else being equal, reduce home cooking time [dH<0] and increase swimming time [dLp>0]).[1]

This aspect of the framework implies that energy expenditure (E) increases only if the utility associated with additional investment in some physical activity (e.g., swimming, Lp) rises. Budget constraints mean that the investment necessary for overweight individuals to achieve their ideal weight (W_0) must compete with other (i.e., sedentary) activities that offer higher utility. This factor suggests that financial incentives ought to be targeted at activities where the opportunity cost of physical activity is the lowest.

People choose resource allocations that maximize their utility (U) subject to resource constraints (Y and Z) such that the opportunity cost of time allocated to leisure (L), which increases utility directly, are:

(1) sleep (S) and home (H) activities that increase utility directly ;

(2) work (O), which does not affect utility directly, but provides income (Y) for expenditure in other domains ($L, $T, $H);

(3) travel (T), which increases with distance (mO, mL) traveled to work and leisure facilities; decreases with speed; and typically provides modest utility (e.g., car drivers may enjoy their in-car entertainment systems, whereas cyclists may enjoy being outside); or even dis-utility (e.g., the frustration arising from unpredictable traffic congestion).

Impact of Financial Incentives

Financial incentives are interpreted as increasing or decreasing the cost of given activities. Sufficient reduction in the price of swimming (d$Lp<0), for example, alters the utility-maximizing allocation of resources for some individuals and encourages more swimming. However, the impact in overall energy expenditure (E) is complex and unpredictable unless more information about personal preferences (including their valuation of future weight βv [W']) and willingness to trade one activity for another is taken into account. Consider just two types of people proposed by Yaniv.[5] First, the financial incentive might encourage nonswimmers ("non-health-conscious people") to start swimming at the expense of sedentary leisure activities (a substitution effect). But second, the financial incentive

simply increases the income (Y) of existing swimmers ("health conscious people" who place a high value on βv [W']). If they also cycle to work, they might be inclined to respond by increasing travel expenditure ($T) in order to get to work faster by switching to sedentary travel modes (the income effect). Although both types of people have benefited from the financial incentive (in terms of overall utility), energy expenditure (E) only increases in the first case. In the second, it might fall. These alternative scenarios are explored in Table A2.

Although their impact seems ambiguous, financial incentives may be most useful for encouraging physical activity in non-health-conscious people (defined as above) because (for them) the opportunity cost of additional physical activity is always sedentary activity (so E unambiguously increases). Of course, this assumes that they are actually persuaded to forgo their sedentary activities. So the remaining question is: How large does the incentive need to be?

A financial incentive for active leisure (d$Lp) requires a payment that offsets the difference between utility losses from sedentary activities (e.g., watching less TV, dLs<0) and utility gains arising from more physical activity (e.g., more swimming, dLp>0):

$$d\$Lp > \left[\frac{dU}{dLs}\ dLs + \frac{dU}{dLp}\ dLp\right] \tag{VII}$$

Consider non-health-conscious people who may place little value on their future weight (βv [W']) and may gain very little direct utility from active leisure (e.g. , swimming). The incentive payment (d$Lp) must reimburse forgone sedentary leisure activities (e.g., watching TV, Ls), which are of greater value than an equal allocation of time to active leisure (e.g., swimming, Lp). According to the "last-hour rule," the opportunity cost of sedentary leisure activities is equal to utility associated with any other activity, including work. In order to change behavior, the incentive might need to be relatively large, perhaps equivalent to the amount they are paid at work (i.e., the wage rate per unit of time, $O).

In contrast, an active travel financial incentive requires a payment that reimburses the difference in an individual's valuation of forgone sedentary travel (dTs < 0) and new active travel (dTp > 0):

$$d\$Tp > \left[\frac{dU}{dTs}\ dTs + \frac{dU}{dTp}\ dTp\right] \tag{VIII}$$

This active travel incentive could be much smaller than the active leisure incentive (d$Tp < d$Lp) in some cases. First, consider a "non-health-conscious" individual who works reasonably near home so that active travel is viable in terms of distance, but who currently always drives. Noting that their drive to work provides minimal utility directly (compared to sedentary leisure) but access to work facilities, the opportunity cost of sedentary travel is relatively small because active travel also allows them equal accessibility. In this framework, the only losses arise if sedentary modes are slower, so that the time taken to travel increases (dT>0), resulting in forgone O, L, and H, or are less comfortable (although this may be negligible for short urban journeys). Individuals may also save money if active travel is cheaper than sedentary travel ($Ts –$Tp). Second, even if the financial incentive does not increase the energy expenditure in health -conscious people who are already very active in their leisure time, these individuals would gain utility if they substitute active travel for active leisure and use the additional time and income to enjoy more-expensive (sedentary) leisure activities.

Table A1. Summary of simplifying assumptions

Domain	Time allocated to domain (in the short-term)	Physical activity (in the short-term)	Rationale and other assumptions
Sleep	Variable	Fixed–None	Hours of sleep are not affected by changes in other (time, money) resource allocations or physical activity.
Leisure	Variable	Variable	
Occupation	Fixed	Fixed	At least in the short-term, job, wage, working hours, and work and home locations (and therefore distance traveled) are fixed (although in the longer term, people make choices about their job and work hours as with any other decision in the economic framework).<br>Wages cannot be saved in one period for spending in another period.
Transport	Variable (in terms of speed and therefore time), but distance traveled (mO and mL) is fixed for given activities	Variable	Distance travelled to leisure activities is determined by the quality of local facilities (which are fixed, at least in the short-term).<br>The time and expenditure investment required to travel a given distance varies by travel mode (sedentary travel is likely to be more expensive and, in many cases, faster).<br>Time allocated to active travel has a similar impact on energy expenditure and weight as time allocated to active leisure.
Home	Fixed	Variable	

Note: In the long-term, all domains are variable (e.g., people can move home and change their working hours; and better leisure facilities might open locally), but for the purposes of analyzing the impact of financial incentives for active travel and active leisure, the economic rational-choice framework described in the Appendix makes the simplifying assumptions shown in the table. mO and mL=distance from home to any specific work (mO) or leisure (mL) activity (e.g., a person's own workplace, or their preferred gym).

Table A2. How the actual impact of financial incentives may deviate from the expected or desired impact

Financial incentive policy to promote:		Active leisure	Active travel	Healthy eating (an example from[5])
Example		Free swimming lessons	Free bikes	Thin subsidy
Desired impact	On relative prices	Reduction in relative price of physical leisure activities (d$Lp<0)	Reduction in relative price of active travel (d$Tp<0)	Reduction in relative price of healthy food
	On utility-maximization position	U (last hour of active leisure) > U (last hour of other activities)	U (last hour of active travel) > U (last hour of other activities)	U (last hour of home cooking) > U (last hour of other activities)
	On W'	Increase in energy expenditure (E) and decrease in W'	Increase in energy expenditure (E) and decrease in W'	Decrease in food consumption (F) and decrease in W'
Example of actual impact on behavior of 'health-conscious people' (i.e., people with low fast-food food consumption and high exercise consumption)	Income effect	If swim already, then more income to spend on other activities (perhaps sedentary [e.g., Ts])<br>Decrease in E	If cycle (to work or leisure) already, then more income to spend on other activities (perhaps sedentary [e.g., Ls])<br>Decrease in E	If home cook already, then more income to spend on other activities (perhaps sedentary, e.g., [Ls])<br>Decrease in E
	Substitution effect	If swim already, then may swim more often at the expense of other sedentary or physical activities<br>No change or an increase in E	If cycle already, then may increase length of existing journeys at the expense of other sedentary travel or other activities<br>No change or an increase in E	May cook more healthy food, which is time-consuming and sedentary, at the expense of other physical activities<br>Decrease in E and F
Example of actual impact on behavior of 'non health-conscious people' (i.e., people with high junk-food consumption and low energy expenditure)	Income	Not applicable	Not applicable	Not applicable
	Substitution effect	If not a swimmer, then may swim more often at the expense of other sedentary leisure activities<br>Increase in E	If not a cyclist, then may cycle more often at the expense of other sedentary travel<br>Increase in E	If not a cook, then may eat more healthy food instead of junk food, using time at the expense of other sedentary activities<br>Decrease in F
Empirical Evidence		Limited[6]	See Evidence Synthesis section in the main paper	More widely studied

References for Appendix A

1. Cawley J. An economic framework for understanding physical activity and eating behaviors. Am J Prev Med 2004;27(3 Suppl 1):117–25.
2. Frank LD. Economic determinants of urban form: resulting trade-offs between active and sedentary forms of travel. Am J Prev Med 2004;27(3):146–53.
3. Pratt M, Macera CA, Sallis JF, O'Donnell M, Frank LD. Economic interventions to promote physical activity: Application of the SLOTH model. Am J Prev Med 2004;27(3, Suppl 1):136–45.
4. Lakdawalla D, Philipson T. The growth of obesity and technological change. Econ Hum Biol 2009;7(3):283–93.
5. Yaniv G, Rosin O, Tobol Y. Junk-food, home cooking, physical activity and obesity: the effect of the fat tax and the thin subsidy. J Public Econ 2009;93(5-6):823–30.
6. Fordham R, Barton G. A cost effectiveness scenario analysis of four interventions to increase child and adolescent physical activity: the case of walking buses, free swimming, dance classes and community sports. London: NICE, Promotion of Physical Activity in Children Programme Guidance, 2008.
